# Selective Synaptic Remodeling in Rat Auditory and Visual Cortices Following Noise‐Induced Permanent Hearing Loss in Adulthood

**DOI:** 10.1155/np/8852865

**Published:** 2026-05-18

**Authors:** Miao Zhao, Yanjie Bai, Yaxin Yang, Na Li, You Zhou

**Affiliations:** ^1^ Laboratory of Sensory Neurobiology, School of Basic Medical Sciences, Hebei University, Baoding, Hebei Province, China, hbu.cn; ^2^ Key Laboratory of Aging and Health in Hebei Province, School of Basic Medical Sciences, Hebei University, Baoding, Hebei Province, China, hbu.cn

**Keywords:** A1 cortex, cross-modal plasticity, hearing loss, sensory reorganization, V1 cortex

## Abstract

In adults with acquired hearing loss, cortical reorganization and cross‐modal plasticity critically influence the timing and outcomes of cochlear implantation. This study examined the effects of 2 weeks of bilateral auditory deprivation—induced by intense broadband noise—on the structure and gene expression in the primary auditory (A1) and visual (V1) cortices of adult rats. The results revealed that the total neuronal counts in both the A1 and V1 remained unchanged compared to controls. Morphological analysis showed no significant change in mature dendritic spine density in either region, but immature spine density was significantly reduced in the V1 cortex. Ultrastructural examination demonstrated notable synaptic alterations in the A1 cortex, including significant reductions in postsynaptic density (PSD) length, thickness, area, and synaptic vesicle number—changes that were not observed in the V1 cortex. Transcriptomic profiling indicated a region‐specific response, with 197 differentially expressed genes (DEGs) in the A1 cortex, compared to a broader alteration of 545 DEGs in the V1 cortex, which were significantly involved in neuronal signaling and synaptic transmission. These findings demonstrate that auditory deprivation induces distinct molecular and synaptic remodeling in the A1 and V1 cortices, identifying neuronal plasticity and synaptic regulation as central mechanisms for cross‐modal reorganization in the adult brain.

## 1. Introduction

Neuroplasticity refers to the brain’s remarkable ability to adapt to internal or external stimuli by altering its structure, function, or connectivity. This adaptive capacity allows organisms to make lasting changes in response to environmental interactions [1]. In sensory and motor systems, the lack of experience or use leads to the shrinkage of cortical representations associated with underutilized systems or limbs. While plasticity is most pronounced during critical periods of early development, it continues into adulthood, though in a more limited capacity [2]. In the adult brain, sensory systems retain a degree of plasticity, enabling adaptation to new experiences, recovery from injury, and compensation for sensory loss. For example, the auditory system adapts to hearing loss through homeostatic plasticity. Cortical and subcortical plasticity in the auditory system following auditory deprivation during adulthood is well‐documented. These changes extend beyond the critical developmental period, but the underlying molecular mechanisms require further investigation [3–5].

Cross‐modal plasticity represents another form of neuroplasticity, where the brain enhances the function of other senses when one sensory modality is impaired. This phenomenon is well‐documented in both developmental and adult models of sensory deprivation [6]. For instance, blind individuals often exhibit enhanced abilities in sound localization, pitch discrimination [7–9], tactile acuity [10, 11], and odor identification [12, 13]. These improvements are accompanied by profound neural plasticity in the brain. Cross‐modal plasticity may arise from the reorganization of the deprived cortex to process information from spared senses. It also involves adaptive changes in the spared cortices [14–17]. Similarly, cross‐modal reorganization occurs in clinical populations with hearing loss, including congenital deafness, sudden deafness in adulthood, and age‐related hearing loss. Growing evidence suggests that auditory deprivation leads to the recruitment of the auditory cortex for processing other sensory information, such as visual or somatosensory inputs [18]. Despite these advancements, the functional roles, neural networks, and molecular mechanisms underlying cross‐modal plasticity in the cerebral cortex and subcortical structures remain areas of active research.

Cross‐modal plasticity is widely regarded as a fundamental mechanism that enables the brain to adapt to environmental changes. It serves as a natural self‐repair system and a beneficial adaptation. However, this plasticity can also complicate the recovery of specific lost sensory functions. For example, auditory deprivation is often associated with poorer hearing outcomes following cochlear implantation. This is mainly due to the extensive reorganization of cortical and subcortical structures that result from prolonged auditory deprivation. In adult postlingual deaf patients undergoing cochlear implantation, the outcomes are significantly influenced by the age at which deafness occurred and the duration of auditory deprivation [19, 20].

In this study, we utilized a rat model of intense noise‐induced hearing loss to investigate the effects of a 2‐week period of bilateral auditory deprivation on neural plasticity in both the auditory and visual cortices. Our aim is to provide deeper insights into the molecular mechanisms underlying cross‐modal plasticity in adulthood. Furthermore, this research seeks to elucidate the potential compensatory changes in visual processing that may occur in individuals with hearing loss. These findings may offer valuable implications for the development of effective rehabilitation strategies.

## 2. Methods and Materials

### 2.1. Subjects

Sprague‐Dawley rats (adult male, 200–220 g) were obtained from SiPeiFu (Beijing) Biotechnology Co., Ltd. (Beijing, China) and maintained under a 12/12 h light/dark cycle, with standard food and water provided ad libitum. All rats were divided randomly into noise‐exposed and sham control groups and were housed in the same room with low ambient acoustical noise (<50 dB SPL) except for the noise exposure and audiometry. In all experiments, rats were deeply anesthetized with an intraperitoneal injection of pentobarbital sodium (80 mg/kg) prior to all terminal procedures. For perfusion‐based experiments, animals were transcardially perfused with saline followed by 4% paraformaldehyde (PFA) under deep anesthesia. Brains were then collected. For acute tissue collection, rats were euthanized by decapitation under deep anesthesia, and the brains were immediately removed. The experimental procedures were approved by the Ethics Committee of Hebei University and were performed in accordance with the guideline for experimental animal welfare of Hebei University (HBU2024M027). This study was designed, conducted, and reported in compliance with the ARRIVE guidelines. All efforts were made to minimize the number of animals used and their suffering.

### 2.2. Noise Exposure

Acoustic overexposure was conducted in a double‐walled sound‐attenuating chamber (Guangdong Shengzuo Acoustics Co., Ltd.). Rats were placed in a standard cage and were able to freely move during the duration of the exposure to noise. A JBL CS673 speaker was placed facing down on top of the cage’s lid, with an 8–20 kHz bandpass noise generated by the RZ6 workstation (Tucker‐Davis Technologies, Alachua, FL, USA). Noise exposure was performed at 120–122 dB SPL for 2 h via an amplifier (SAST SA‐5016) to induce permanent threshold shifts (PTSs). The control rats spent the same amount of time in the sound‐attenuating chamber but did not receive noise exposure. Auditory brainstem responses (ABRs) were recorded before noise and sham exposures and at day 14 post noise and sham exposures.

### 2.3. ABRs

ABRs were recorded in a sound‐attenuating chamber (Guangdong Shengzuo Acoustics Co., Ltd., China) following a previously published protocol [21]. Rats were anesthetized with intraperitoneal injection of pentobarbital sodium (80 mg/kg), and body temperature was maintained at 37°C using an isothermal pad (Homeothermic Monitoring System, Harvard Apparatus) placed under the abdomen. Three subdermal needle electrodes were positioned at the vertex (active), right mastoid region (reference), and left shoulder (ground). Acoustic stimuli were generated by the RZ6 system (Tucker‐Davis Technologies) and delivered through an MF1 speaker positioned approximately 10 cm above the vertex. Tone bursts (3 ms duration, 1 ms rise/fall) were presented in half‐octave steps from 45 to 4 kHz, with sound levels decreasing from 90 to 0 dB SPL in 5 dB steps. Stimulus were delivered at a rate of 20 per second, and 400 responses were averaged at each frequency and each level. ABR thresholds were defined as the lowest stimulus level eliciting any of the initial four waveform peaks. Near‐threshold recordings were repeated to confirm the results.

### 2.4. Immunofluorescent Staining

Rats were deeply anesthetized with pentobarbital sodium (80 mg/kg, i.p.). Cochleae were carefully removed and postfixed in 4% PFA, then decalcified in 10% EDTA‐Na_2_. After decalcification, the basilar membranes were dissected, permeabilized with 1% Triton X‐100, and subsequently blocked for 1 h at room temperature. The tissues were then incubated overnight at 4°C with the following primary antibodies: polyclonal rabbit anti‐Myosin7α (25‐6790, Proteus Bioscience) diluted 1:300 in 5 % serum. After three washes with PBS, the samples were incubated with the corresponding secondary antibodies for 1–2 h at room temperature in the dark. Immunolabeled images were captured using a laser scan confocal fluorescent microscope (LSM710; Carl Zeiss, Jena, Germany).

### 2.5. Nissl Staining

Rats were deeply anesthetized with pentobarbital sodium (80 mg/kg, i.p.) and perfused with saline followed 4% PFA. After brain extraction, the tissues were postfixed in formaldehyde and dehydrated using a sucrose gradient. Once dehydration was complete, the brains were frozen and sectioned coronally (16 μm) using Leica VT1000S vibrating blade microtome (Leica, Germany) to obtain slices containing the A1 or V1 cortex. The sections were air‐dried at room temperature, stained with Cresyl violet solution (Solarbio, China) for 15 min, and rinsed with deionized water. Differentiation was performed in Nissl differentiation solution (Solarbio, China) for several seconds up to 2 min, with microscopic monitoring until the background was nearly colorless. Sections were then rapidly dehydrated in absolute ethanol, cleared in xylene, and mounted with neutral resin. Images were captured using an upright microscope (Nikon, Sendai, Japan).

### 2.6. Golgi Staining

Rats were deeply anesthetized with pentobarbital sodium (80 mg/kg, i.p.). The brain fixed with PFA is subjected to frozen sectioning to obtain tissue sections containing the A1 or V1 cortex (150 μm). Golgi staining was carried out FD Rapid GolgiStain Kit (PK401, FD NeuroTechnologies, Columbia, USA), according to the manufacturer’s instructions. The tissue sections were imaged utilizing a Zeiss LSM confocal microscope (Carl Zeiss, Germany) equipped with a ×63 oil‐immersion objective lens. The basal and apical oblique dendrites of L2/3 pyramidal neurons in A1 and V1 cortices were imaged. For dendritic spine density and maturity analysis, images were blinded, and spines were counted using ImageJ and manually sorted as previously described [22].

### 2.7. Transmission Electron Microscope (TEM)

Following anesthesia with pentobarbital sodium (80 mg/kg, i.p.) and transcardial perfusion with saline, rat brains were rapidly extracted, and 1 mm^3^ tissue blocks were dissected from the Layer II/III of primary cortices. The tissue blocks were fixed in 2% glutaraldehyde and 1% osmium tetroxide for 2 h at 4°C, followed by sequential dehydration through an ethanol gradient series. The ethanol was then replaced with propylene oxide as a transitional solvent prior to embedding in Epon 812 resin. Serial ultrathin sections were prepared (80 nm) using an ultramicrotome equipped with a diamond knife. These sections were subsequently contrasted with lead citrate and examined using a Philips CM‐120 TEM (Philips, Eindhoven, Netherlands). Quantitative ultrastructural analysis was performed using Image J software (NIH, USA), with the following parameters measured: (1) postsynaptic density (PSD) dimensions (length, width, and area), (2) synaptic cleft width, (3) synaptic vesicle density (number/μm^2^), and (4) synaptic curvature.

### 2.8. Transcriptome

Rats were deeply anesthetized with pentobarbital sodium (80 mg/kg, i.p.), and brains ere rapidly extracted. Coronal brain sections containing the primary cortices were obtained using a vibratome. The A1 and V1 cortices were precisely isolated based on anatomical landmarks, including the hippocampus, medial geniculate body, and rhinal sulcus. For each region, three cDNA libraries were constructed from control (Con) and noise‐exposure (NE) rats, following Illumina protocols (Shanghai Majorbio Co., China). Transcriptome experiments and subsequent data analysis were conducted according to previously published methods [23]. Library concentration was quantified using a Qubit 4.0 fluorometer, and sequencing was performed on the Illumina NovaSeq 6000 platform using the NovaSeq Reagent Kit. Raw sequencing data were stored in FASTQ format. Raw paired‐end reads were processed for trimming and quality control using fastp with default settings. Clean reads were then aligned to the reference genome using HISAT2. Transcript assembly for each sample was performed with StringTie in a reference‐guided manner. Gene expression levels were quantified as transcripts per million reads (TPM). Differentially expressed genes (DEGs) were identified using the criteria of fold change ≥1.5 and *p*  < 0.05 (Tables S1 and S2). Functional annotation of DEGs was performed using the Kyoto Encyclopedia of Genes and Genomes (KEGG) and Gene Ontology (GO) databases.

To ensure more stringent control of false discoveries, the DEG analysis in the supplementary transcriptomic dataset was re‐performed using adjusted *p*‐values rather than unadjusted *p*‐values. The DEGs identified based on adjusted *p*‐values, together with the enrichment results and their corresponding descriptions, are presented in Figures S1–S5 and Tables S8–S13.

### 2.9. Quantitative PCR (qPCR)

Following anesthesia with pentobarbital sodium (80 mg/kg, i.p.), rat brains were rapidly excised, and tissue samples from the A1 cortex and V1 cortex were precisely dissected using a vibrating microtome. Total RNA was extracted using Trizol (Sangong Biotech, Shanghai, China) according to the manufacturer’s protocol. The synthesis of first‐strand cDNA and qPCR was performed using PrimeScript RTase and SYBR Premix ExTaq Kit (Takara, Dalian, Hebei, China). Primers were synthesized by GenScript (Nanjing, Jiangsu, China) and the sequences of them were listed in Table S3. β‐Actin and GAPDH were used as endogenous controls. The relative expression levels were calculated by the 2^−*ΔΔ*CT^ method.

### 2.10. Statistical Analysis

Sample size was determined a priori with reference to published studies employing comparable paradigms and outcome measures in adult rodent models of noise‐induced hearing loss and cortical plasticity and was further supported by an a priori power analysis performed in 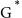 Power (v3.1.9.7) for the primary two‐group comparisons (two‐sided independent‐samples *t* test; *α* = 0.05; power = 0.80). Using an effect size estimated from pilot/observed group means and standard deviations (Cohen’s *d* = 2.38), the minimum required sample size was *n* = 4–5 rats per group; accordingly, *n* = 5 independent biological replicates (rats) per group were used for all key analyses. For histological quantifications, multiple sections and fields acquired from the same animal were treated as technical replicates and were averaged to yield a single value per animal prior to statistical testing, thereby avoiding pseudoreplication. Data are presented as the mean±SEM and were analyzed using GraphPad Prism 7 (GraphPad Software, La Jolla, CA, USA). For comparisons between two groups, an unpaired two‐tailed Student’s *t*‐test was used for normally distributed data, whereas the Mann–Whitney *U* test was used when data did not meet the assumptions of normality. *p*  < 0.05 was considered to be statistically significant.

## 3. Results

### 3.1. Two Weeks of Auditory Deprivation Does Not Alter Neuron Counts in A1 and V1 Cortices

**Figure 1:**
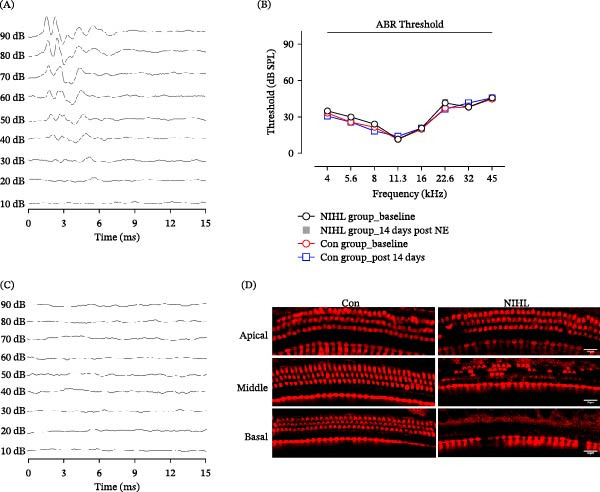
Intensity noise exposure induced permanent hearing loss. (A, B) Representative ABR waveforms at 16 kHz before noise exposure and 14 days after noise exposure in rats. (C) Auditory threshold curves show a permanent threshold shift 14 days after noise exposure, with thresholds exceeding 90 dB SPL across all tested frequencies (gray lines indicate thresholds >90 dB) (*n* = 5 rats per group). (D) Immunofluorescence staining of the cochlea (scale bar = 20 µm) shows significant hair cell loss (Myosin7α‐positive), particularly in outer hair cells, 14 days after noise exposure compared to control rats without noise exposure.

**Figure 2:**
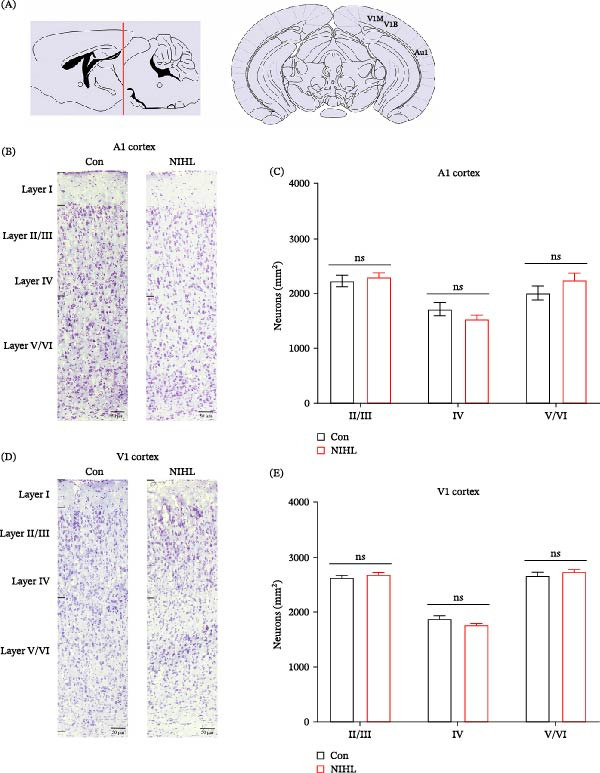
Neuron counts in the primary cortices before and after noise exposure. (A) Anatomical locations of the A1 and V1 cortices in the rat brain. A representative coronal section at Bregma −3.27 mm (interaural 0.52 mm) is shown. The schematic was hand‐drawn based on the Paxinos and Watson rat stereotaxic atlas, with the approximate boundaries of A1 and V1 used for subsequent analyses indicated. (B, D) Representative Nissl stained micrographs of neurons in the A1 and V1 cortices from control and NIHL rats. Scale bar = 50 µm. (C) Quantification of neuronal density (number of neurons per unit cortical layer area) in the A1 cortex revealed no significant difference between NIHL (red bars) and control (black bars) groups (*n* = 5 rats per group). (E) Similar quantification of neuronal density in the V1 cortex showed no significant difference between NIHL and control groups (*n* = 5 rats per group). Data are presented as mean±SEM. Statistical significance was assessed using Student’s *t*‐test.

To evaluate the impact of auditory deprivation on hearing sensitivity, ABR measurements were obtained before and 2 weeks after exposure to high‐intensity broadband noise. The results showed a profound and irreversible elevation in hearing thresholds exceeding 90 dB across all tested frequencies, confirming the successful induction of permanent hearing loss. Histological analysis revealed significant loss of cochlear hair cells, particularly outer hair cells, predominantly in the basal and middle turns of the cochlea (Figure 1). Nissl staining of brain tissue sections was used to quantify neuronal density in the A1 and V1 cortices. Two weeks following noise exposure, neuronal counts in both cortical regions remained statistically unchanged compared to control animals (Figure 2). These findings indicate that short‐term, complete auditory deprivation does not significantly impact gross neuronal survival in either the A1 or V1 cortex.

### 3.2. Two Weeks of Auditory Deprivation Alters Synaptic Structure in A1 and V1 Cortices

**Figure 3:**
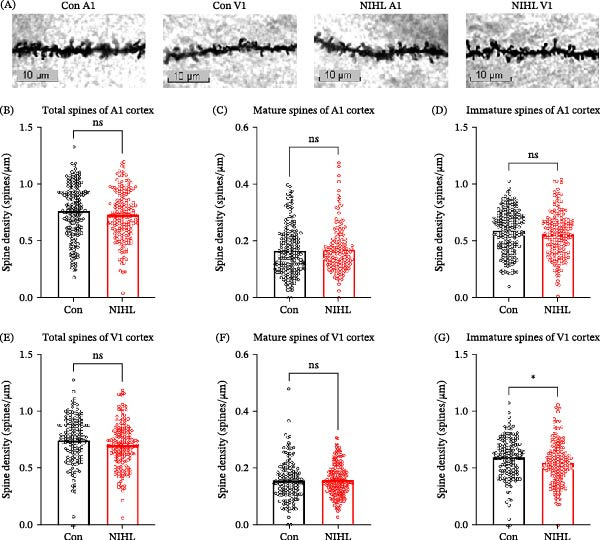
Dendritic spine density in the primary cortices before and after noise exposure. (A) Representative micrographs of neuronal dendrites in the primary auditory cortex (A1) and primary visual cortex (V1) from control and noise‐induced hearing loss (NIHL) groups. Scale bar = 10 µm. More than 15 neurons were analyzed per group from five rats (*n* = 5 rats per group; ≥15 neurons/group). (B–D) Quantification of (B) total spine density, (C) mature spine density, and (D) immature spine density in the A1 cortex. (E–G) Corresponding quantification in the V1 cortex: (E) total spine density, (F) mature spine density, and (G) immature spine density (*p* = 0.0276). Data are presented as mean±SEM. Statistical analysis was performed using the unpaired Student’s *t*‐test; *p*  < 0.05 was considered statistically significant.

**Figure 4:**
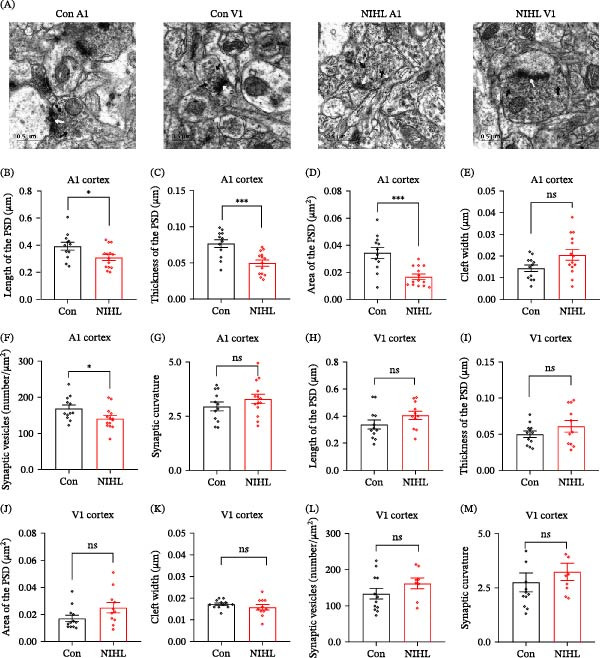
Synaptic ultrastructure in the primary cortices before and after noise exposure. (A) Representative TEM image of synaptic ultrastructure in the primary cortices before and after noise exposure. Scale bar = 0.5 μm. (B–G) Quantitative analysis of PSD length (*p* = 0.0258), PSD thickness (*p* = 0.0005), PSD area (*p* = 0.0003), cleft width (*p* = 0.0506), synaptic vesicles density (*p* = 0.0349), and synaptic curvature (*p* = 0.2459) in the A1 cortex. (H–M) Quantitative analysis of PSD length (*p* = 0.1454), PSD thickness (*p* = 0.2308), PSD area (*p* = 0.0709), cleft width (*p* = 0.2880), synaptic vesicles density (*p* = 0.2085), and synaptic curvature(*p* = 0.2188) in the V1 cortex. Data are presented as mean±SEM, *n* = 5 rats per group. Statistical analysis was performed using the unpaired Student’s *t*‐test and Mann–Whitney *U* test; *p*  < 0.05 was considered statistically significant.

To investigate synaptic and dendritic plasticity following auditory deprivation, we performed Golgi staining and dendritic spine density and morphology. In the A1 cortex, no significant differences were observed between control and NE animals in total dendritic spine density, or in the densities of mature and immature spines (Figure 3A–D). Similarly, in the V1 cortex, total spine density and the density of mature spines remained unchanged. However, a significant reduction in immature spine density was observed in the V1 cortex of noise‐exposed animals (Figure 3E–G), suggesting a potential disruption in synaptic development or remodeling within the visual cortex, possibly reflecting compensatory processes or synaptic stress rather than adaptive plasticity. TEM further revealed region‐specific ultrastructural synaptic alterations. In the A1 cortex, noise‐exposed rats showed significant reductions in PSD length, thickness, and area, along with decreased synaptic vesicle density per square micrometer, compared to controls (Figure 4A–D,F). However, synaptic cleft width and synaptic curvature were not significantly altered (Figure 4E,G). In contrast, no significant changes were detected in any synaptic parameters examined in the V1 cortex (Figure 4H–M). These data suggest that auditory deprivation leads to synaptic degeneration and weakened synaptic transmission in the A1 cortex, while synaptic structure in the V1 cortex remains largely preserved, potentially reflecting either compensatory reorganization or synaptic stress in response to the auditory deprivation rather than plasticity aimed at functional adaptation.

### 3.3. Auditory Deprivation Differentially Alters Gene Expression Profiles in A1 and V1 Cortices

**Figure 5:**
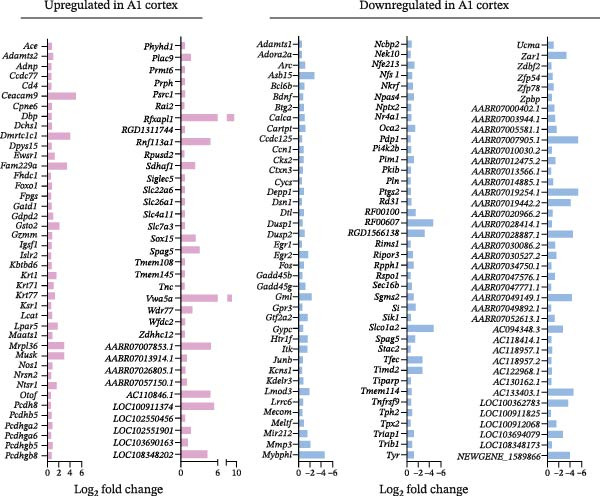
Up‐ and downregulated genes in the A1 cortex. A total of 77 upregulated genes in the A1 cortex are shown in red bars, while 120 downregulated genes are shown in blue bars.

**Figure 6:**
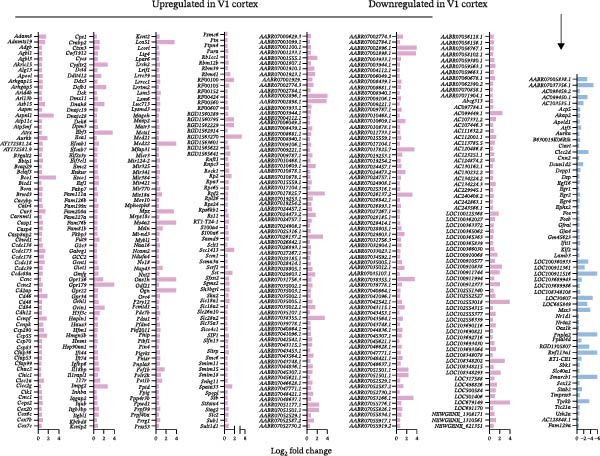
Up‐ and downregulated genes in the V1 cortex. A total of 487 upregulated genes in the V1 cortex are shown in red bars, while 58 downregulated genes are shown in blue bars.

**Figure 7:**
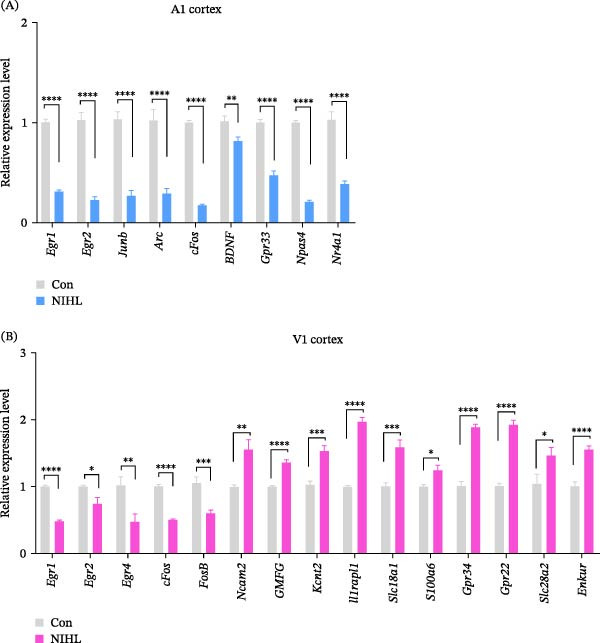
Validation of DEGs identified by transcriptomic analysis using qPCR. (A) In the A1 cortex, nine DEGs were validated by qPCR and showed significant downregulation consistent with the transcriptomic results. Among them, *Egr1*, *Junb*, *Arc*, *BDNF*, and *Nr4a1* met the *q*‐value criterion, whereas *Egr2*, *cFos*, *Gpr3*, and *Npas4* were selected based on the *p*‐value criterion only. (B) In the V1 cortex, 15 DEGs were validated by qPCR and exhibited directionally concordant up‐ or downregulation with the transcriptomic data. *Egr1*, *Egr2*, *Egr4*, *FosB*, *Ncam2*, *Kcnt2*, *Il1rapl1*, *Gpr34*, *Gpr22*, *Slc28a2*, and *Enkur* met the *q*‐value criterion, whereas *cFos*, *GMFG*, *Slc18a1*, and *S100a6* were selected based on the *p*‐value criterion only. Data are presented as mean±SEM. Unpaired Student’s *t*‐test:  ^∗^ *p*  < 0.05,  ^∗∗^ *p*  < 0.01,  ^∗∗∗^ *p*  < 0.001,  ^∗∗∗∗^ *p*  < 0.0001.

Transcriptomic analysis revealed distinct and region‐specific gene expression changes in response to 2 weeks of auditory deprivation. In the A1 cortex, 77 genes were significantly upregulated and 120 genes were downregulated (Figure 5 and Table S1). In contrast, the V1 cortex exhibited a more pronounced transcriptomic response, with 487 genes upregulated and 58 genes downregulated (Figure 6 and Table S2), suggesting a higher degree of molecular plasticity and compensatory reorganization in this region. qPCR was conducted to validate a subset of DEGs identified by RNA‐Seq (Figure 7). In the A1 cortex, nine DEGs—including Egr, Junb, and Arc—were validated. In the V1 cortex, fifteen DEGs were confirmed, including Egr, c‐Fos, and Ncam, supporting the reliability of the transcriptomic data and highlighting region‐specific molecular responses to auditory deprivation.

### 3.4. Functional Enrichment Analysis of DEGs in the A1 Cortex

**Figure 8:**
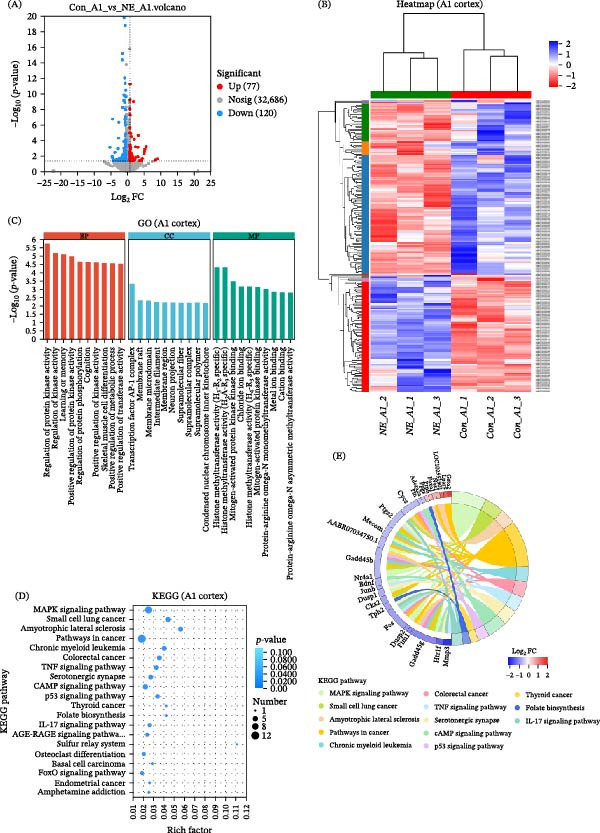
GO and KEGG analysis of DEGs in the A1 cortex. (A) Volcano plot showing the DEGs in the A1 cortex. (B) Heatmap of 197 DEGs, including 77 upregulated and 120 downregulated genes. (C) GO enrichment analysis of biological processes associated with the upregulated and downregulated genes. The numbers on the right indicate the number of genes in each category. The top 10 enriched GO terms are shown. (D) KEGG pathway enrichment analysis, highlighting signaling pathways with different biological functions. The numbers on the right indicate the number of genes involved in each pathway. The top 20 enriched pathways are presented. (E) Chord diagram illustrating the top DEGs with the largest log_2_ FC and their association with the most significantly enriched KEGG pathway (lowest *p*‐value). Detailed GO and KEGG analysis results are provided in Tables S4 and S5.

GO enrichment analysis was conducted to explore the biological processes (BPs) associated with DEGs in each cortical region. In the A1 cortex, significantly enriched BP terms included regulation of protein kinase activity, protein phosphorylation, modulation of chemical synaptic transmission, regulation of calcium ion transport, and biosynthetic process regulation. In the molecular function (MF) category, DEGs were associated with transcription regulator activity, RNA polymerase II regulatory region sequence‐specific DNA binding, active ion transmembrane transporter activity, and anion transmembrane transporter activity. Cellular component (CC) terms were enriched for neuronal structure, including neuron projection, neuronal cell body, axon, dendrite, synapse, and extracellular matrix. KEGG pathway analysis further identified significant enrichment in pathways associated with neuron signaling and plasticity, including the MAPK, TNF, p53, and cAMP signaling pathways. Additionally, the serotonergic synapse pathway was enriched, indicating potential involvement of neurotransmitter‐mediated mechanisms (Figure 8 and Tables S4 and S5). These findings suggest that short‐term auditory deprivation elicits a multifaceted molecular response in the A1 cortex involving synaptic remodeling, transcriptional regulation, and intracellular signaling linked to neuronal function, plasticity, and stress adaptation.

### 3.5. Functional Enrichment Analysis of DEGs in the V1 Cortex

**Figure 9:**
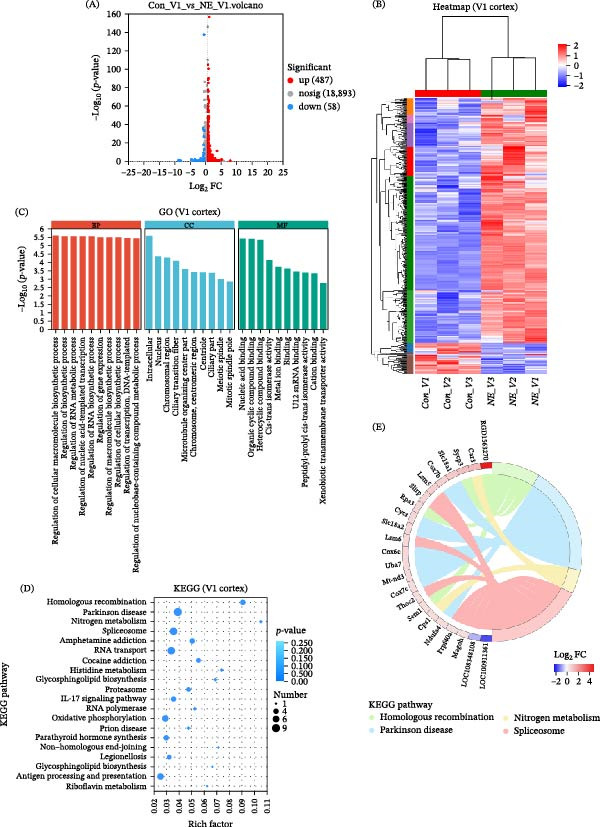
GO and KEGG analysis of DEGs in the V1 cortex. (A) Volcano plot showing the DEGs in the V1 cortex. (B) Heatmap of 545 DEGs, including 487 upregulated and 58 downregulated genes. (C) GO enrichment analysis of biological processes associated with the upregulated and downregulated genes. The numbers on the right indicate the number of genes in each category. The top 10 enriched GO terms are shown. (D) KEGG pathway enrichment analysis, highlighting signaling pathways with different biological functions. The numbers on the right indicate the number of genes involved in each pathway. The top 20 enriched pathways are presented. (E) Chord diagram illustrating the top DEGs with the largest log_2_ FC and their association with the most significantly enriched KEGG pathway (lowest *p*‐value). Detailed GO and KEGG analysis results are provided in Table S6 and Table S7.

In the V1 cortex, GO enrichment analysis of DEGs revealed broad involvement in transcriptional regulation, neural development, and cellular structure organization. In the BP category, highly enriched terms included regulation of RNA metabolic process, gene expression, nucleic acid–templated transcription, and DNA–templated transcription. Processes related to neuronal development and synaptic organization—such as neuron projection arborization, axon extension involved in axon guidance, dendrite arborization, central nervous system development, and basal dendrite morphogenesis—were also significantly enriched. Additionally, several DEGs were implicated in circadian regulation, chromatin organization, and response to neurotransmitters and hormonal stimuli (e.g., response to kainic acid and response to growth hormone), indicating dynamic transcriptional and signaling plasticity in the V1 cortex. In the CC category, DEGs were enriched in nuclear and chromosomal structure—including the nucleus, chromosomal region, condensed chromosome, and telomere cap complex, as well as neuronal and cytoskeletal components such as neuron projection, dendrite, axon, the microtubule organizing center, mitotic spindle, and synaptic membrane. Additionally, enrichment of terms such as ciliary part, periciliary membrane compartment, and nonmotile cilium suggests potential alterations in intracellular transport mechanisms and signaling microdomains. In the MF category, DEGs in the V1 cortex were significantly associated with transcriptional and epigenetic regulation, including transcription regulator activity, core promoter sequence‐specific DNA binding, histone serine kinase activity, and chromatin binding. Additional enriched functions involved ion channel and neurotransmitter regulation, such as sodium channel activity, chloride‐activated potassium channel activity, and intracellular sodium‐activated potassium channel activity. Moreover, DEGs linked to hormone and neuropeptide signaling—such as retinoid receptor binding, calcitriol (active vitamin D) binding, and neuropeptide interaction—indicate that hormonal signaling pathways may play a role in modulating cortical plasticity in response to sensory deprivation. KEGG pathway analysis of DEGs in the V1 cortex identified significant enrichment in four pathways: homologous recombination, Parkinson’s disease, RNA transport, and nitrogen metabolism. While the number of enriched pathways was limited compared to the A1 cortex, the identified pathways provide important insight into the molecular processes underlying cross‐modal plasticity (Figure 9 and Tables S6 and S7). In particular, the involvement of RNA transport and chromatin‐related pathways suggests heightened transcriptional and epigenetic activity in the visual cortex as it adapts to the loss of auditory input.

## 4. Discussion

Hearing loss induces clinically significant sensory deprivation, driving extensive anatomical and physiological reorganization throughout the central auditory system. The nature and extent of cortical plasticity depend on three critical factors: the severity of the hearing impairment (ranging from mild to profound deafness) [24], the developmental timing of deprivation onset (congenital/early‐onset versus adulthood) [25, 26], and the duration of auditory deprivation [27, 28]. Notably, sensory deprivation during the developmental period elicits heightened neural plasticity, facilitating more substantial functional reorganization in the brain. Both human and animal studies demonstrate that early‐onset profound deafness triggers sensory replacement, with the deprived auditory cortex being progressively recruited for visual and tactile processing [29–32]. This cross‐modal plasticity likely mediates the observed behavioral enhancements in visual perception among the congenitally deaf [25]. Although adult neural plasticity is more limited than during development, the mature brain retains adaptive capacity. Prolonged auditory deprivation in adulthood can trigger compensatory mechanisms, such as enhanced processing in non‐auditory regions like the visual cortex. However, these processes differ from those seen after early‐life deprivation. Recent studies in adult rats revealed that long‐term auditory deprivation (3 months) causes intermodal processing imbalances, characterized by altered excitatory/inhibitory markers in auditory cortex and increased visual cortex activity [27]. In contrast, the effects of short‐term adult auditory deprivation remain poorly understood. Our study demonstrates that 2 weeks of auditory deprivation in adult rats, induced by intense broadband noise exposure, triggers region‐specific synaptic and transcriptomic changes in both auditory and visual cortices. Despite no gross neuronal loss, this permanent hearing loss led to synaptic degeneration in the auditory cortex and transcriptional remodeling in both regions. These findings offer new insights into cross‐modal plasticity and cortical adaptation in the adult brain, with clinical implications for optimizing cochlear implantation timing and rehabilitation strategies.

### 4.1. Sensory Deprivation and Cortical Stability in Adulthood

Our results demonstrated that 2 weeks of profound bilateral auditory deprivation did not significantly alter neuronal density in either the A1 or V1 cortices. These findings support the hypothesis that cortical neuron survival in the adult brain is relatively resilient to short‐term sensory deprivation. This observation aligns with established literature indicating that neuroplasticity in adults primarily involves synaptic reorganization and connectivity changes rather than neurogenesis or apoptosis [33, 34]. Supporting this interpretation, a recent study investigating parvalbumin (PV) and perineuronal net (PNN) expression in A1 following noise exposure, which reported preserved PV^+^/PNN^+^ cell density but identified layer‐ and cell type‐specific reductions in PNN fluorescence intensity [35]. These modifications may disrupt excitatory‐inhibitory balance stabilizing into pathological configurations. The absence of neuron loss in our study strongly implies that functional deficits and compensatory mechanisms arise primarily from alterations in synaptic‐level adaptation, following short‐term auditory deprivation. Consistent with this view, our ultrastructural analyses revealed region‐specific pathology: A1 exhibited pronounced synaptic degeneration characterized by reductions in PSD size, thickness, and surface area, coupled with decreased synaptic vesicle density. These morphological alterations likely underlie impaired synaptic transmission efficiency and may explain observed auditory processing deficits.

At the molecular level, transcriptomic profiling provided mechanistic insights into these phenomena. Immediate‐early genes (IEGs), established markers of neuronal activity, are recognized as components of the generalized neuronal response to natural stimuli. Previous studies have demonstrated neuronal and synaptic plasticity in the auditory cortex through comparative analysis of IEG expression dynamics [36, 37]. Given their established correlation with sensory‐evoked neuronal activity in auditory cortex, IEGs typically show transient but rapid downregulation following auditory deprivation [38, 39]. Our findings reveal significant downregulation of several IEGs (including c‐Fos, Arc/Arg3.1, Egr 1, and Egr2) persisting through longitudinal deafness (2 weeks post profound hearing loss). However, the present study did not assess potential recovery of IEGs downregulation. Previous studies have confirmed that prolonged auditory deprivation leads to a recovery or increasing trend in the expression levels of these IEGs [36, 37], strongly suggesting time‐dependent plasticity characteristics in the auditory cortex following sensory deprivation. Notably, brain‐derived neurotrophic factor (BDNF), a neuropeptide critical for synaptic development and activity‐dependent synaptic plasticity [40], showed significant reduction in A1 cortex 2 weeks post profound hearing loss. This finding corroborates prior reports demonstrating that bilateral cochlear ablation in adult rats substantially decreases BDNF and its receptor Tropomyosin receptor Kinase B levels in auditory cortex [41]. KEGG pathway analysis revealed alterations in MAPK and cAMP signaling pathways in the A1 cortex following 2‐week auditory deprivation. The MAPK pathway contributes to long‐term synaptic plasticity by potentiating BDNF transcriptional activation after auditory stimulation [42], while cAMP regulates Arc/Arg3.1 expression [43]. Our data also identified changes in TNF‐mediated homeostatic synaptic plasticity, with TNF signaling modifications emerging after 2‐week deprivation. Existing evidence implicates neuroplasticity‐related genes (including Junb, Edn1, and Ptgs2) in auditory deafferentation through TNF pathway regulation [44], suggesting potential TNF‐mediated modulation of Junb and Ptgs2 in A1 cortex postauditory deprivation.

Our transcriptomic data suggest that gene expression decreases and synaptic remodeling in the A1 cortex following auditory deprivation are likely regulated by MAPK, cAMP, and TNF signaling pathways. Other affected pathways, such as p53 signaling, serotonergic synapse, and IL‐17 signaling, require further investigation. In contrast, while V1 showed no ultrastructural changes, a significant reduction in immature dendritic spine density was observed. This selective change may reflect synaptogenesis and pruning rebalancing, indicating targeted synaptic refinement in the visual cortex in response to auditory input loss.

### 4.2. Cross‐Modal Plasticity: Compensatory Changes in the Visual Cortex

One of the key findings of this study is the extensive transcriptomic reorganization in the V1 cortex, despite no direct injury or structural degeneration. Compared to the A1 cortex, the V1 cortex showed a more robust molecular response, with over 480 upregulated and 58 downregulated genes following auditory deprivation. Functional enrichment analyses highlighted involvement in transcriptional regulation, chromatin remodeling, axon guidance, dendritic arborization, and intracellular signaling—processes critical to experience‐dependent plasticity. This transcriptional activity suggests adaptive remodeling in the V1 cortex in response to auditory input loss, a hallmark of cross‐modal plasticity. Previous studies in both human and animal models have documented enhanced visual processing following hearing loss, including increased visual activation of auditory areas, improved visual attention, and enhanced visual motion perception [24, 25, 32, 45, 46]. Our findings add to this body of literature by identifying underlying molecular and synaptic mechanisms, including reduced immature spine density, enriched gene expression related to neuronal development, and altered ion channel function. These changes may reflect an ongoing shift in cortical resource allocation, whereby the brain compensates for auditory deprivation by reinforcing visual processing pathways. Clinically, such cross‐modal reorganization poses a double‐edged sword. While it provides a form of functional compensation in sensory‐deprived individuals, it may also complicate rehabilitation. Specifically, in patients undergoing cochlear implantation after prolonged deafness, the auditory cortex may become functionally repurposed for processing visual or somatosensory inputs, thereby reducing its availability for auditory perception once hearing is restored. This phenomenon has been observed in several clinical studies, where excessive visual recruitment of the auditory cortex correlates with poorer post‐implantation speech perception [47–49].

### 4.3. Temporal Windows, Molecular Targets, and Therapeutic Implications

The current findings also highlight the importance of timing in the context of intervention for adult‐onset hearing loss. Although cross‐modal plasticity is often considered a developmental phenomenon, this study shows that significant transcriptional and synaptic changes can occur within just 2 weeks of auditory deprivation in the adult brain. This rapid onset of cortical remodeling underscores the narrow window during which therapeutic interventions, such as cochlear implantation or auditory training, may be most effective. Our data suggest that even short‐term auditory deprivation initiates synaptic weakening in A1 and compensatory changes in V1. This aligns with prior research indicating that longer durations of deafness are associated with greater cross‐modal reorganization and worse auditory rehabilitation outcomes [19, 24, 47]. The enrichment of pathways related to synaptic transmission, transcriptional regulation, and neuronal signaling in both cortices suggests an early molecular response, potentially preceding more pronounced functional and anatomical reorganization. Thus, early intervention after hearing loss—even in adulthood—could prevent or mitigate maladaptive plasticity and improve long‐term outcomes. Reactivating auditory pathways through electrical stimulation or targeted cognitive training may preserve auditory cortical function and prevent excessive cross‐modal takeover. Additionally, identifying early biomarkers of cortical reorganization, such as the DEGs identified here, could help personalize treatment timing and intensity, enhancing patient‐specific outcomes.

The transcriptomic profiling conducted in this study reveals potential molecular targets for therapeutic modulation. In the A1 cortex, DEGs were enriched in pathways including MAPK, TNF, p53, and cAMP signaling—pathways commonly implicated in synaptic plasticity, neuroinflammation, and cellular stress responses. These pathways may be amenable to pharmacological intervention aimed at preserving synaptic integrity and supporting auditory processing after sensory loss. Conversely, the gene expression profile in the V1 cortex emphasized transcriptional regulation, chromatin remodeling, and hormone signaling. Interestingly, the identification of enriched terms related to retinoid receptor binding and vitamin D activity suggests that endocrine modulation may influence cross‐modal plasticity. This raises the possibility that hormonal or nutritional interventions could be developed to modulate sensory compensation and support balanced cortical plasticity following sensory deprivation. The involvement of neuropeptide‐related genes and neurotransmitter signaling in the V1 cortex may inform strategies to modulate excitability and connectivity in spared sensory cortices, enhancing or controlling compensatory responses following auditory deprivation.

## 5. Conclusion

In summary, this study demonstrates that short‐term auditory deprivation in adult rats induces rapid and region‐specific molecular and synaptic remodeling in both auditory and visual cortices. These findings underscore the remarkable neuroplasticity of the adult brain in response to sensory loss, and highlight the critical role of timing in auditory rehabilitation. The first systematic characterization of distinct molecular pathways underlying cortical remodeling opens new avenues for developing targeted therapies to enhance or modulate neuroplasticity following sensory deprivation. However, several limitations and future research directions should be considered. First, longitudinal studies are needed to track the progression of cross‐modal plasticity and determine whether these changes stabilize or become irreversible over time. Second, although the noise‐induced hearing loss model effectively mimics certain forms of human hearing impairment (e.g., occupational or trauma‐related hearing loss), it does not fully capture age‐related or idiopathic deafness. Future studies should explore whether similar plastic changes occur across different etiologies of hearing loss and whether therapeutic interventions can reverse or redirect maladaptive cortical reorganization. Finally, expanding the scope of analysis to include higher‐order brain regions, along with functional imaging and behavioral assessments, will be essential to elucidate how molecular and synaptic adaptations influence perceptual and cognitive outcomes. By addressing these gaps, future research may provide a more comprehensive understanding of sensory deprivation‐induced plasticity and its implications for rehabilitation strategies.

## Author Contributions

Conceptualization, data curation, methodology, formal analysis: Miao Zhao, Yanjie Bai, Yaxin Yang, and Na Li. Investigation, writing, supervision, funding acquisition: You Zhou.

## Funding

This study was funded by the Science and Technology Research Project of Universities in Hebei Province (Grant QN2024198) and the Medical Science Foundation of Hebei University (Grant 2022A04).

## Disclosure

All authors have read and agreed to the published version of the manuscript. After using ChatGPT, the authors have reviewed and edited the content as needed and take full responsibility for the content of the publication.

## Conflicts of Interest

The authors declare no conflicts of interest.

## Supporting Information

Additional supporting information can be found online in the Supporting Information section.

## Supporting information


**Supporting Information** In addition to the main content of this manuscript, supporting materials have been provided to enhance the understanding and rigor of our study. These include: Table S1: Gene expression patterns and DEGs in the A1 cortex. Table S2: Gene expression pattern and DEGs in V1 cortex. Table S3: The primers used for qPCR verification. Table S4: GO analysis of DEGs in A1 cortex. Table S5: KEGG analysis of DEGs in the A1 cortex. Table S6: GO analysis of DEGs in V1 cortex. Table S7: KEGG analysis of DEGs in V1 cortex. Table S8: Gene expression pattern and DEGs in A1 cortex (DEGs were screened using an adjusted *p*‐value). Table S9: Gene expression pattern and DEGs in V1 cortex (DEGs were screened using an adjusted p‐value). Table S10: GO analysis of DEGs in A1 cortex (DEGs were screened using an adjusted p‐value). Table S11: KEGG analysis of DEGs in A1 cortex (DEGs were screened using an adjusted p‐value). Table S12: GO analysis of DEGs in V1 cortex (DEGs were screened using an adjusted p‐value). Table S13: KEGG analysis of DEGs in V1 cortex (DEGs were screened using an adjusted *p*‐value). These supporting files contain critical data sets and analyses that were integral to our research findings. Figure S1: The up‐ and downregulated genes in the A1 cortex based on adjusted p‐value screening, corresponding to Table S8. Figure S2: The up‐ and downregulated genes in the V1 cortex based on adjusted p‐value screening, corresponding to Table S9. Figure S3: qPCR validation of selected DEGs identified from the adjusted p‐value‐based transcriptomic results. Figure S4: GO and KEGG enrichment analyses of adjusted p‐value‐screened DEGs in the A1 cortex, corresponding to Tables S10 and S11. Figure S5: GO and KEGG enrichment analyses of adjusted p‐value‐screened DEGs in the V1 cortex, corresponding to Tables S12 and S13.

## Data Availability

The data that support the findings of this study are available from the corresponding author upon reasonable request.
