# Supplementary material for: Selective Synaptic Remodeling in Rat Auditory and Visual Cortices Following Noise‐Induced Permanent Hearing Loss in Adulthood
Source: Neural Plast. 2026 May 18;2026:8852865. doi: 10.1155/np/8852865 (PMC13182752; doi:10.1155/np/8852865)
Supplement: Supplementary file 1 — Supporting Information In addition to the main content of this manuscript, supporting materials have been provided to enhance the understanding and rigor of our study. These include: Table S1: Gene expression patterns and DEGs in the A1 cortex. Table S2: Gene expression pattern and DEGs in V1 cortex. Table S3: The primers used for qPCR verification. Table S4: GO analysis of DEGs in A1 cortex. Table S5: KEGG analysis of DEGs in the A1 cortex. Table S6: GO analysis of DEGs in V1 cortex. Table S7: KEGG analysis of DEGs in V1 cortex. Table S8: Gene expression pattern and DEGs in A1 cortex (DEGs were screened using an adjusted p‐value). Table S9: Gene expression pattern and DEGs in V1 cortex (DEGs were screened using an adjusted p‐value). Table S10: GO analysis of DEGs in A1 cortex (DEGs were screened using an adjusted p‐value). Table S11: KEGG analysis of DEGs in A1 cortex (DEGs were screened using an adjusted p‐value). Table S12: GO analysis of DEGs in V1 cortex (DEGs were screened using an adjusted p‐value). Table S13: KEGG analysis of DEGs in V1 cortex (DEGs were screened using an adjusted p‐value). These supporting files contain critical data sets and analyses that were integral to our research findings. Figure S1: The up‐ and downregulated genes in the A1 cortex based on adjusted p‐value screening, corresponding to Table S8. Figure S2: The up‐ and downregulated genes in the V1 cortex based on adjusted p‐value screening, corresponding to Table S9. Figure S3: qPCR validation of selected DEGs identified from the adjusted p‐value‐based transcriptomic results. Figure S4: GO and KEGG enrichment analyses of adjusted p‐value‐screened DEGs in the A1 cortex, corresponding to Tables S10 and S11. Figure S5: GO and KEGG enrichment analyses of adjusted p‐value‐screened DEGs in the V1 cortex, corresponding to Tables S12 and S13. [file NP-2026-8852865-s001.zip › 8852865.f1/Supplementary Information-4.9.docx]

**3.3 Auditory deprivation differentially alters gene expression profiles in A1 and V1 cortices**

Transcriptomic analysis revealed distinct, region-specific gene expression changes in response to two weeks of auditory deprivation. In the A1 cortex, 6 genes were significantly upregulated and 16 genes were downregulated (Figure 5, Table S8). In contrast, the V1 cortex exhibited a more pronounced transcriptomic response, with 359 genes upregulated and 24 genes downregulated (Figure 6, Table S9), suggesting a higher degree of molecular plasticity and compensatory reorganization in this region. Quantitative PCR was conducted to validate a subset of DEGs identified by RNA-Seq (Figure 7). In the A1 cortex, five DEGs—including Junb, Arc, and BDNF—were validated. In the V1 cortex, eleven DEGs were confirmed, including Egr, FosB, and Ncam, supporting the reliability of the transcriptomic data and highlighting region-specific molecular responses to auditory deprivation.

**3.4 Functional Enrichment Analysis of DEGs in the A1 Cortex**

GO enrichment analysis was conducted to explore the biological processes associated with DEGs in each cortical region. In the A1 cortex, significantly enriched Biological Process (BP) terms included were related to learning or memory, cognition, learning, behavior, response to calcium ion, positive regulation of cell differentiation and cell growth, regulation of developmental growth, and response to metal ion. In the Molecular Function (MF) category, DEGs were associated with DNA-binding transcription activator activity (RNA polymerase II-specific) and regulatory region sequence-specific DNA binding. Cellular Component (CC) terms were enriched for neuronal and synaptic structures, including neuron projection, neuron part, presynaptic membrane, synaptic membrane, glutamatergic synapse, postsynaptic endosome, and transcription factor AP-1 complex. KEGG pathway analysis further revealed significant enrichment in signaling and neuromodulatory pathway, notably the TNF, MAPK, IL-17, and cAMP signaling pathways, as well as serotonergic synapse and retrograde endocannabinoid signaling (Figure 8, Table S10-S11). These findings suggest that short-term auditory deprivation elicits a multifaceted molecular response in the A1 cortex involving synaptic remodeling, transcriptional regulation, and intracellular signaling linked to neuronal function, plasticity, and stress adaptation.

**3.5 Functional Enrichment Analysis of DEGs in the V1 Cortex**

In the V1 cortex, GO enrichment analysis indicated that the V1 response was dominated by regulation of gene expression and biosynthetic program. In the Biological Process domain, enriched terms were primarily related to regulation of cellular and macromolecule biosynthetic processes, regulation of nucleobase containing compound metabolism, regulation of DNA templated transcription, regulation of RNA metabolic and biosynthetic processes, regulation of nucleic acid templated transcription, and regulation of gene expression. In the Cellular Component domain, enrichment was concentrated in chromosomal and cytoskeletal structures and intracellular compartments, including chromosome, microtubule, microtubule organizing center, intracellular organelle, intrinsic component of synaptic membrane, and respiratory chain complex IV. In the Molecular Function domain, enriched functions were mainly associated with nucleic acid binding and broad small molecule binding activities, including heterocyclic compound binding, organic cyclic compound binding, metal ion binding, and cation binding, with additional enrichment for U12 snRNA binding and a smaller set of ion channel related activities.

KEGG pathway analysis yielded a limited set of significantly enriched pathway, with the strongest signals in RNA transport, homologous recombination, and Parkinson disease. Taken together, these results suggest that, under q value control, the V1 cortex engages a coherent transcriptional program characterized by intensified regulation of transcriptional output, altered intracellular structural and mitochondrial associated components, and pathway level enrichment consistent with changes in RNA handling, genome maintenance, and cellular stress adaptation (Figure 9, Table S12-S13).

**Figure 5. Up- and downregulated genes in the A1 cortex.** A total of 6 upregulated genes in the A1 cortex are shown in red bars, while 16 downregulated genes are shown in blue bars.

**
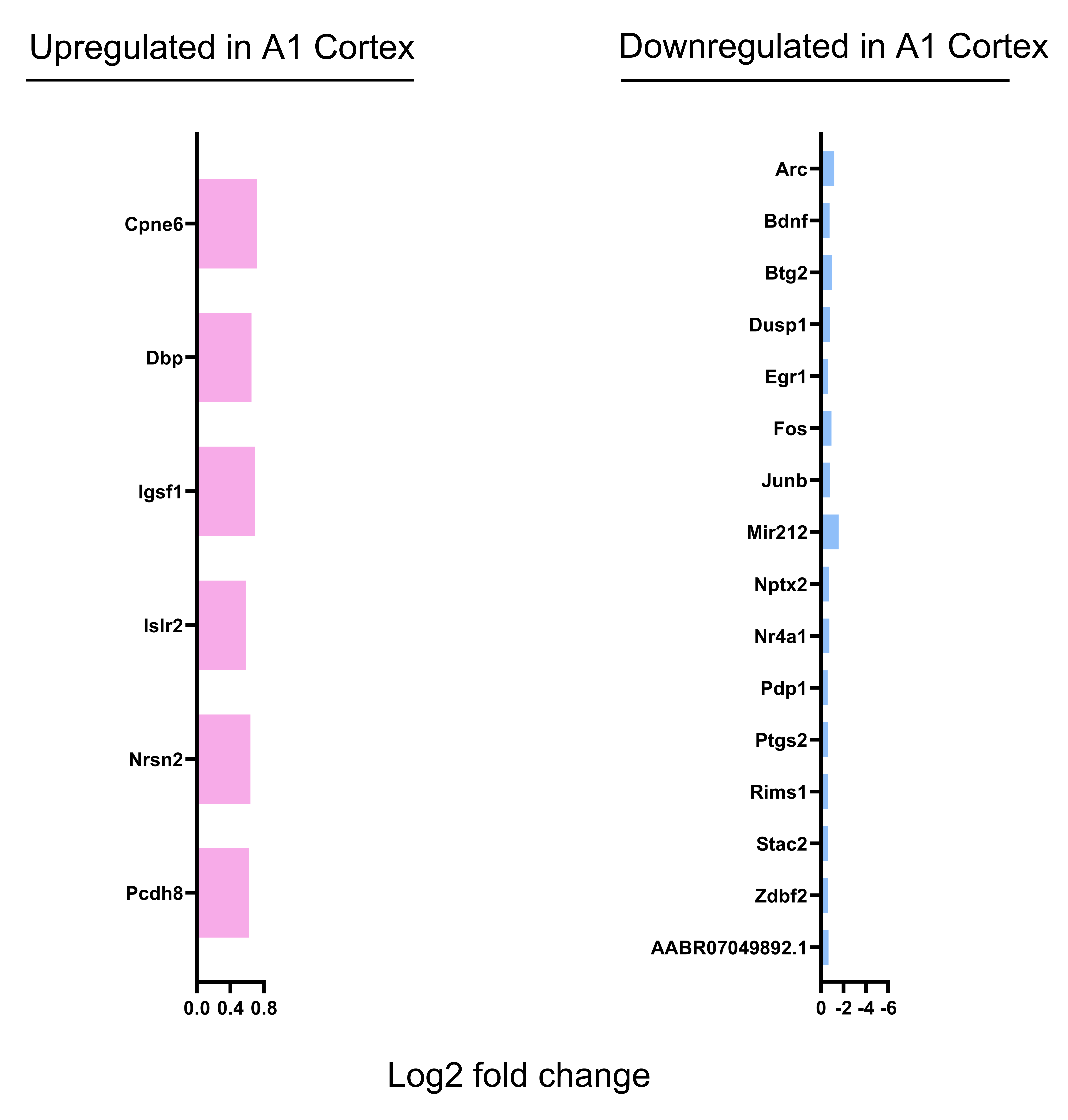
**

**Figure 6. Up- and downregulated genes in the V1 cortex.** A total of 359 upregulated genes in the V1 cortex are shown in red bars, while 24 downregulated genes are shown in blue bars.

**
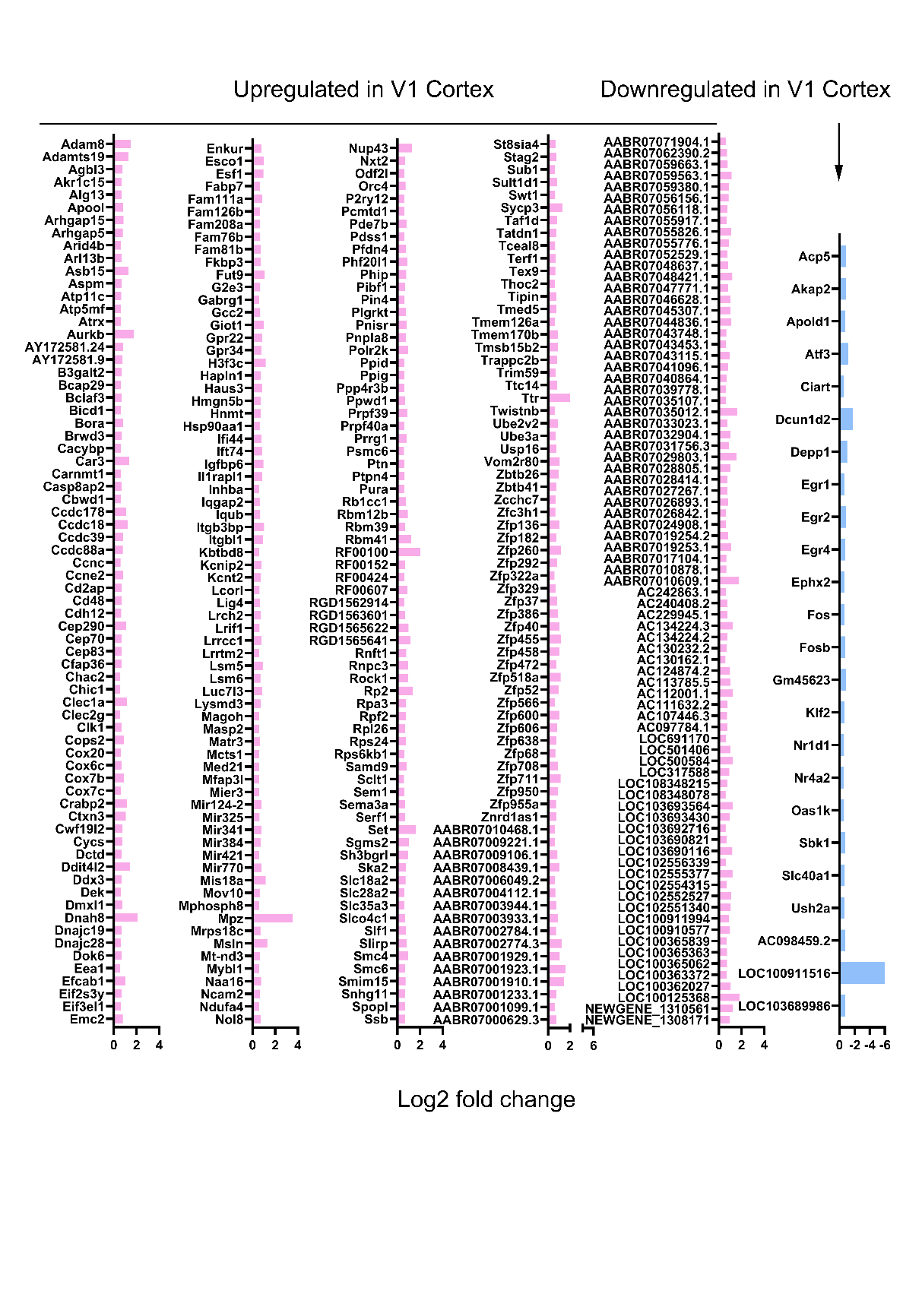
**

**Figure 7. Validation of DEGs identified by transcriptomic analysis using qPCR.** (A) The expression levels of five selectively validated DEGs were significantly downregulated in the A1 cortex, consistent with the transcriptomic data. (B) The expression levels of eleven selectively validated DEGs were significantly upregulated or downregulated in the V1 cortex, also consistent with the transcriptomic results. Data are presented as mean ± SEM. Unpaired Student’s t-test: *p < 0.05, **p < 0.01, ***p < 0.001, ****p < 0.0001.

**
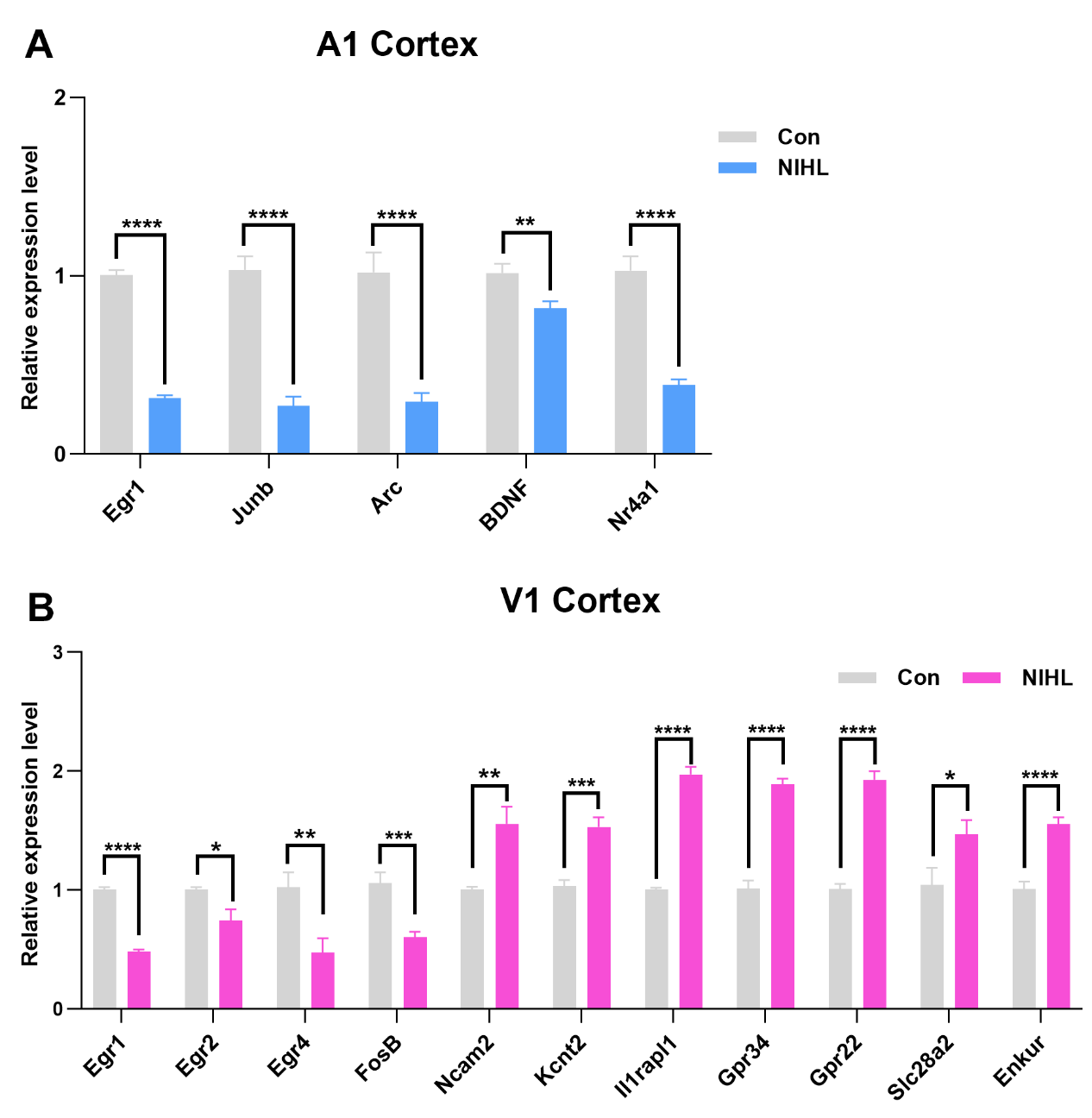
**

**Figure 8.** **GO and KEGG analysis of DEGs in the A1 cortex.** (A) Volcano plot showing the DEGs in the A1 cortex. (B) Heatmap of 22 DEGs, including 6 upregulated and 16 downregulated genes. (C) GO enrichment analysis of biological processes associated with the upregulated and downregulated genes. The numbers on the right indicate the number of genes in each category. The top 10 enriched GO terms are shown. (D) KEGG pathway enrichment analysis, highlighting signaling pathways with different biological functions. The numbers on the right indicate the number of genes involved in each pathway. The top 20 enriched pathways are presented. (E) Chord diagram illustrating the top DEGs with the largest logFC and their association with the most significantly enriched KEGG pathway (lowest q-value). Detailed GO and KEGG analysis results are provided in Table S10 and Table S11.

**Figure 9. GO and KEGG analysis of DEGs in the V1 cortex.** (A) Volcano plot showing the DEGs in the V1 visual cortex. (B) Heatmap of 383 DEGs, including 359 upregulated and 24 downregulated genes. (C) GO enrichment analysis of biological processes associated with the upregulated and downregulated genes. The numbers on the right indicate the number of genes in each category. The top 10 enriched GO terms are shown. (D) KEGG pathway enrichment analysis, highlighting signaling pathways with different biological functions. The numbers on the right indicate the number of genes involved in each pathway. The top 20 enriched pathways are presented. (E) Chord diagram illustrating the top DEGs with the largest logFC and their association with the most significantly enriched KEGG pathway (lowest q-value). Detailed GO and KEGG analysis results are provided in Table S12 and Table S13.
