# Supplementary material for: Selective Synaptic Remodeling in Rat Auditory and Visual Cortices Following Noise‐Induced Permanent Hearing Loss in Adulthood
Source: Neural Plast. 2026 May 18;2026:8852865. doi: 10.1155/np/8852865 (PMC13182752; doi:10.1155/np/8852865)
Supplement: Supplementary file 1 — Supporting Information In addition to the main content of this manuscript, supporting materials have been provided to enhance the understanding and rigor of our study. These include: Table S1: Gene expression patterns and DEGs in the A1 cortex. Table S2: Gene expression pattern and DEGs in V1 cortex. Table S3: The primers used for qPCR verification. Table S4: GO analysis of DEGs in A1 cortex. Table S5: KEGG analysis of DEGs in the A1 cortex. Table S6: GO analysis of DEGs in V1 cortex. Table S7: KEGG analysis of DEGs in V1 cortex. Table S8: Gene expression pattern and DEGs in A1 cortex (DEGs were screened using an adjusted p‐value). Table S9: Gene expression pattern and DEGs in V1 cortex (DEGs were screened using an adjusted p‐value). Table S10: GO analysis of DEGs in A1 cortex (DEGs were screened using an adjusted p‐value). Table S11: KEGG analysis of DEGs in A1 cortex (DEGs were screened using an adjusted p‐value). Table S12: GO analysis of DEGs in V1 cortex (DEGs were screened using an adjusted p‐value). Table S13: KEGG analysis of DEGs in V1 cortex (DEGs were screened using an adjusted p‐value). These supporting files contain critical data sets and analyses that were integral to our research findings. Figure S1: The up‐ and downregulated genes in the A1 cortex based on adjusted p‐value screening, corresponding to Table S8. Figure S2: The up‐ and downregulated genes in the V1 cortex based on adjusted p‐value screening, corresponding to Table S9. Figure S3: qPCR validation of selected DEGs identified from the adjusted p‐value‐based transcriptomic results. Figure S4: GO and KEGG enrichment analyses of adjusted p‐value‐screened DEGs in the A1 cortex, corresponding to Tables S10 and S11. Figure S5: GO and KEGG enrichment analyses of adjusted p‐value‐screened DEGs in the V1 cortex, corresponding to Tables S12 and S13. [file NP-2026-8852865-s001.zip › 8852865.f1/Table S3.docx]

**Table S3.** **The primers used for qPCR verification.**

| **Gene Name** | **Direction** | **Sequence (5’-3’)** |
| --- | --- | --- |
| c-fos | F | GTTTCAACGCGGACTACGAGG |
|  | R | GATCTGCGCAAAAGTCCTGTG |
| Arc | F | GAGCGAGAGCTGAAAGGGTT |
|  | R | ACGGTAGAAGACCTCCCTCC |
| JunB | F | CGCAGCTGGGCTTGAGT |
|  | R | CGCTCCTGGTCTTCCATGTT |
| FosB | F | GTGGGCCTTCAACTAGCACA |
|  | R | CCTCCGACGGTTTCTGCATT |
| Egr1 | F | CCTGACCACAGAGTCCTTTTCT |
|  | R | AAAGGGGTTCAGGCCACAAA |
| Egr2 | F | TCGAAAGTACCCCAACAGGC |
|  | R | GTTTCGCATGCAGATCCGAC |
| Egr4 | F | TTCTTCATCCAGGCGGTTCC |
|  | R | GGAGTAAAGGTCCGGCAACA |
| Npas4 | F | CCTCCAAGCAGACTTGAGCA |
|  | R | GCACTGCTTGGTGTCAACTG |
| Nr4a1 | F | CGGTGACGTGCAGCAATTTT |
|  | R | GCTTAGATCGGTAGGCCAGG |
| Kcnt2 | F | TGTGCTTGTGTTGCAGAGAAGG |
|  | R | TGTGCATGGAAAGAAGCATAGGT |
| Bdnf | F | GTCCCGGTATCAAAAGGCCA |
|  | R | ATCCTTATGAACCGCCAGCC |
| Gpr34 | F | CATCGTGGGACTGGTTGGAA |
|  | R | TTATGCGGAAAGGGAGGCAG |
| Gpr22 | F | AACACAGCCACTCCACTGTT |
|  | R | AATGTCATCTCGCACCGTGA |
| Gpr3 | F | TGTTGGTTGCCCTTCACTGT |
|  | R | AAGGCGTAAATGACCGGGTT |
| Il1rapl1 | F | GATCGAATGCAGCGAACTCC |
|  | R | GCCCCTGCTCACTAACATCT |
| Slc28a2 | F | AGAGGCGCTGAAACTGACTG |
|  | R | GGGCCTGAGAAAGAAGGCAT |
| Slc18a1 | F | TGTAAGCTCCCAGCAAGCTC |
|  | R | AGGAGGGATGGTGCCATTTG |
| Ncam2 | F | CAAGCGACAGATGCCAAAGG |
|  | R | TGCTCACTCTGCAAACCACT |
| S100a6 | F | GGTGACAAGCACACCCTGAG |
|  | R | TCCTGATCCTTGTTACGGTCC |
| Gmfg | F | AAAGAGACCAACAATGCCGCC |
|  | R | GGGCTGGAGAAGATGAAACAC |
| Enkur | F | GCAAACGCAATGAGGATGTGA |
|  | R | TCGATGAAGACCGAGAGGGA |
| Gapdh | F | GCAAGTTCAACGGCACAGTCAAG |
|  | R | CGACATACTCAGCACCAGCATCAC |
| β-actin | F | ACTATCGGCAATGAGCGGTTCC |
|  | R | AGCACTGTGTTGGCATAGAGGTC |
